# Defining re-implementation

**DOI:** 10.1186/s43058-023-00440-4

**Published:** 2023-06-05

**Authors:** Rachel Moyal-Smith, James C. Etheridge, Ami Karlage, Yves Sonnay, Christina T. Yuan, Joaquim M. Havens, Mary E. Brindle, William Berry

**Affiliations:** 1 Ariadne Labs, Brigham and Women’s Hospital, Harvard T.H. Chan School of Public Health, 401 Park Drive, 3Rd Floor West, Boston, MA 02215 USA; 2 Department of Surgery, Brigham and Women’s Hospital, Boston, MA USA; 3 Department of Health Policy and Management, Johns Hopkins Bloomberg School of Public Health, Baltimore, MD USA; 4 Department of Surgery, Cumming School of Medicine, University of Calgary, Calgary, Canada; 5 Department of Surgery, Massachusetts General Hospital, Boston, MA USA

**Keywords:** Implementation science, Re-implementation, De-implementation

## Abstract

**Background:**

The first attempt to implement a new tool or practice does not always lead to the desired outcome. Re-implementation, which we define as the systematic process of reintroducing an intervention in the same environment, often with some degree of modification, offers another chance at implementation with the opportunity to address failures, modify, and ultimately achieve the desired outcomes. This article proposes a definition and taxonomy for re-implementation informed by case examples in the literature.

**Main body:**

We conducted a scoping review of the literature for cases that describe re-implementation in concept or practice. We used an iterative process to identify our search terms, pilot testing synonyms or phrases related to re-implementation. We searched PubMed and CINAHL, including articles that described implementing an intervention in the same environment where it had already been implemented. We excluded articles that were policy-focused or described incremental changes as part of a rapid learning cycle, efforts to spread, or a stalled implementation. We assessed for commonalities among cases and conducted a thematic analysis on the circumstance in which re-implementation occurred. A total of 15 articles representing 11 distinct cases met our inclusion criteria. We identified three types of circumstances where re-implementation occurs: (1) failed implementation, where the intervention is appropriate, but the implementation process is ineffective, failing to result in the intended changes; (2) flawed intervention, where modifications to the intervention itself are required either because the tool or process is ineffective or requires tailoring to the needs and/or context of the setting where it is used; and (3) unsustained intervention, where the initially successful implementation of an intervention fails to be sustained. These three circumstances often co-exist; however, there are unique considerations and strategies for each type that can be applied to re-implementation.

**Conclusions:**

Re-implementation occurs in implementation practice but has not been consistently labeled or described in the literature. Defining and describing re-implementation offers a framework for implementation practitioners embarking on a re-implementation effort and a starting point for further research to bridge the gap between practice and science into this unexplored part of implementation.

**Supplementary Information:**

The online version contains supplementary material available at 10.1186/s43058-023-00440-4.

Contributions to the literature

- This article proposes a definition and taxonomy for re-implementation based on case examples in the literature.
- Defining and describing re-implementation provides a unifying terminology and a foundation for future research.
- This article describes the practical implications of re-implementation identified in the literature to guide others in developing their re-implementation strategy.

## Background

The field of implementation science has grown exponentially over the last decade. There has been a proliferation of frameworks, theories, and strategies to support the implementation of evidence-based practices. Even when implementation practitioners appropriately use these in practice, the intended change may not occur, there may be poor adherence, or sites may not be able to sustain changes. Implementation, or the initial process of integrating an intervention within a setting [1], requires an intentional approach with active support and dedicated resources to be effective [2, 3]. Once the change becomes routine and active support and resources end, the sustainability phase begins [3, 4]. However, this is not always the end of the story; implementation may require multiple attempts to obtain the desired outcome.

There are several established process models in implementation science that outline the phases of implementation, with a growing recognition that the implementation process is often non-linear and dynamic, with ongoing adaptations occurring throughout [1, 5–8]. These adaptations are often described as rapid learning cycles or using iterative cycles to test changes on a smaller scale [1, 9], which corresponds to the Plan-Do-Study-Act (PDSA) cycles used in continuous quality improvement [10]. However, there is little detail on the magnitude of change that constitutes a rapid learning cycle, and the dynamism that is described in theory has not yet been translated into clear empirical implications for implementation practice.

There is a need for greater specification of why, when, and how practitioners move *between* implementation phases over time (e.g., moving from the sustainability phase back to an active implementation phase). Lewin’s three-step model of unfreezing, moving, and refreezing, which describes the process of cycling from sustainment to change, offers insight from the change management literature [11]. The first step, unfreezing, disrupts the current equilibrium of a process or behavior, opening the possibility of changing the current state. Movement involves frontline staff and leaders working together to adapt and implement the change. Finally, refreezing the system and making the change part of daily work promotes sustainment and prevents regression to old behaviors. Although a cyclical process of implementation has been described in concept in the implementation science literature [1, 8], it lacks the practical details of moving from sustainability to a more active state found in Lewin’s model.

In addition to moving between the implementation phases, there needs to be more conceptual clarity and empirical descriptions of the magnitude of change and the impact of the degree of change on selecting implementation strategies. In the management literature, the magnitude of change is often described as incremental (i.e., small changes to existing practices) or transformational (i.e., fundamental, qualitative changes) [12]. For example, the punctuated equilibrium model describes long periods of stability (equilibrium) punctuated by bursts of fundamental change (revolution) [13]. During the equilibrium period, there are incremental changes to adapt to changing contexts or address targeted issues [13]. Incremental change is a more common cost-effective approach, requiring little resources while maintaining the stability of the current system [14]. In contrast, the revolutionary period is characterized by changes to a core component, with some form of discontinuity from the initial system. These transformational changes take more time and energy, partly due to overcoming greater resistance to changing a system or process that is already routine [14]. Despite these challenges, transformational change may be necessary after a significant event or when incremental changes reach diminishing returns [14].

The implementation science literature to date has largely focused on describing incremental changes that occur during the adaptation process [10, 15, 16]. In contrast, there has been relatively little attention paid to transformational changes, in which there is a clear break from the status quo, which in turn triggers the need for a greater level of active support. This level of change requires a re-implementation of the intervention, moving from sustainment to implementation, with a similar level of resources as the initial implementation effort. Our years of collective implementation practice have found that sometimes, re-implementation with fundamental changes to the implementation process or intervention is necessary to achieve success. Similarly, unsustained interventions can not simply be turned back on like a light switch but require active support and modifications to adjust to the current context and prevent slippage in the future.

Re-implementation remains largely unexplored. In this article, we propose a definition and taxonomy for re-implementation based on the literature. We describe three distinct types of circumstances in which re-implementation occurs with case examples that provide insights on how understanding each circumstance can inform the implementers’ approach. This debate article aims to define re-implementation and describe its unique considerations for implementation practitioners to adapt their re-implementation strategy and to serve as a foundation for future research in implementation science.

## Methods

We conducted a scoping review to identify cases of re-implementation described in the literature. We chose a scoping review because it aligned with our objective to identify incidences of re-implementation and to clarify or define a concept [17]. Our review followed the five-stage process outlined by Arksey and O’Malley: (1) identifying the research question, (2) identifying relevant studies, (3) study selection, (4) charting the data, and (5) collating, summarizing, and reporting the results [18].

### Identifying the research question

Our study team began by generating a list of questions related to re-implementation, drawing on our experience in practice. Ultimately, we decided we first needed to define the concept. We sought to understand more about re-implementation outside our own experience while focusing on health-related contexts. We identified the following question for our search: 

How has re-implementation been described in concept or practice in the literature?

### Identifying relevant studies

The research team generated a list of potential search terms that were synonyms (re-implement, relaunch, readopt, revive, rebrand, reboot) and phrases that describe re-implementation, such as “implement again” and “repeat implementation.” We then searched for related MESH terms using our list and other root words related to implementation. Next, we pilot-tested these terms using the Google search engine, to determine their prevalence, ways in which they were used, and to identify additional terms. Then, we tested these terms in PubMed and CINAHL, further refining our keywords using an iterative approach, balancing inclusivity with the feasibility of sorting through the results. Our final search terms included the root words re-implement, relaunch, and readopt and excluded specific keywords related to biology and genetics, which use re-implementation to describe genetic sequencing and synthetic biology (Additional file 1). We entered our final search terms into the PubMed and CINAHL databases, which we chose because of our desire to focus on re-implementation in health-related contexts.

### Study selection

**Fig. 1:**
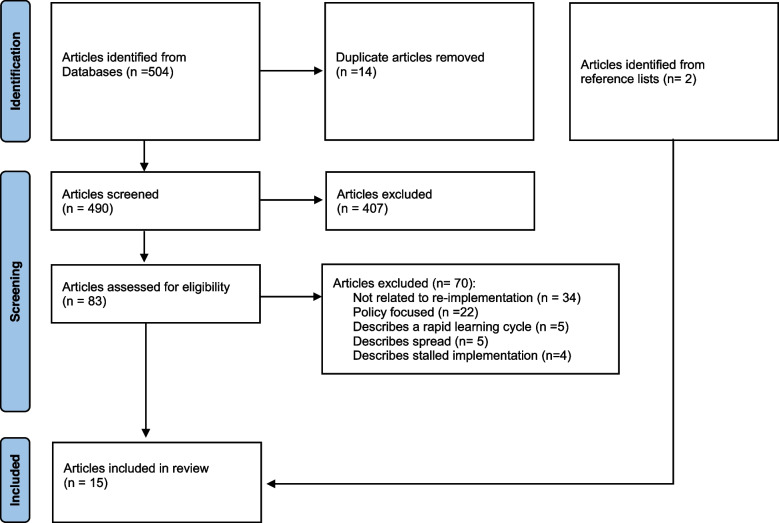
PRISMA flow diagram

We imported the citations from our search into Covidence, a review management tool, for abstract and full-text screening [19]. The study team developed inclusion and exclusion criteria (Table 1) based on implementation practice experience and a preliminary literature review focused on the phases of implementation, including sustainment and de-implementation. Two authors conducted the initial screening of abstracts, reviewing articles for applicability to the study question and the inclusion and exclusion criteria. Due to the lack of evidence base for re-implementation, the two reviewers had a low threshold to discuss articles they were unsure about. Then, one author completed the full-text review, bringing all articles to the study team to discuss their inclusion. Finally, we reviewed the references from the included articles to identify related articles. The PRISMA flow diagram [20] in Fig. 1 details the final number of articles we included and excluded in our review.

**Table 1:** Eligibility criteria

Inclusion criteria	Articles describing or defining implementing an intervention in the same environment where it had already been implemented
Exclusion criteria	Articles describing a rapid learning cycle, spreading an intervention to a new environment, or a stalled implementation. Studies were also excluded if the primary focus was policy or were in a language other than English
Definitions used for eligibility criteria	
Implementation	“Initial process of embedding interventions within settings [1].”
Rapid learning cycle	“Using iterative cycles to test quality improvement changes on a smaller scale [9].”
Spread	Replicating an initiative or intervention in a different environment [21].
Stalled implementation	Implementation effort that is paused or stops making progress [22].

### Charting the data

We developed a table on a shared spreadsheet for data extraction. The table included general information about the article, the intervention that was re-implemented, setting, key findings, and the reason or circumstances that prompted re-implementation. We also included the Expert Recommendations for Implementing Change (ERIC) strategies described in the article, to identify potential commonalities among the cases of re-implementation, and then streamlined these using the ERIC study clusters [23, 24]. The ERIC strategies are a compilation of 73 implementation strategy terms and definitions agreed upon by experts in implementation science.

### Collating, summarizing, and reporting the results

We used inductive thematic analysis to assess commonalities across the selected articles. Although no themes emerged related to the intervention or setting, three themes were identified for the circumstance in which re-implementation occurred. The study team reviewed these themes and cases and if an article fit under more than one theme, the group discussed the dominant circumstances around re-implementation until a consensus was reached.

### Defining re-implementation

We ultimately found 15 relevant articles that represented 11 distinct cases of re-implementation (Additional file 2). Although several cases identified in the literature described or explicitly mentioned re-implementation, we found no formal definition. Based on our review, we propose that re-implementation be defined as the systematic process of reintroducing an intervention in the same environment, often with some degree of modification to either the intervention itself or the implementation strategies used to promote uptake. In these cases, re-implementation began with a comprehensive evaluation after the initial implementation, followed by an intentional strategy to re-launch the intervention. The implementation strategies described varied; however, two ERIC strategy clusters were used in every case: the use of evaluative and iterative strategies and the adaptation and tailoring to context.

### A proposed typology of re-implementation

**Fig. 2:**
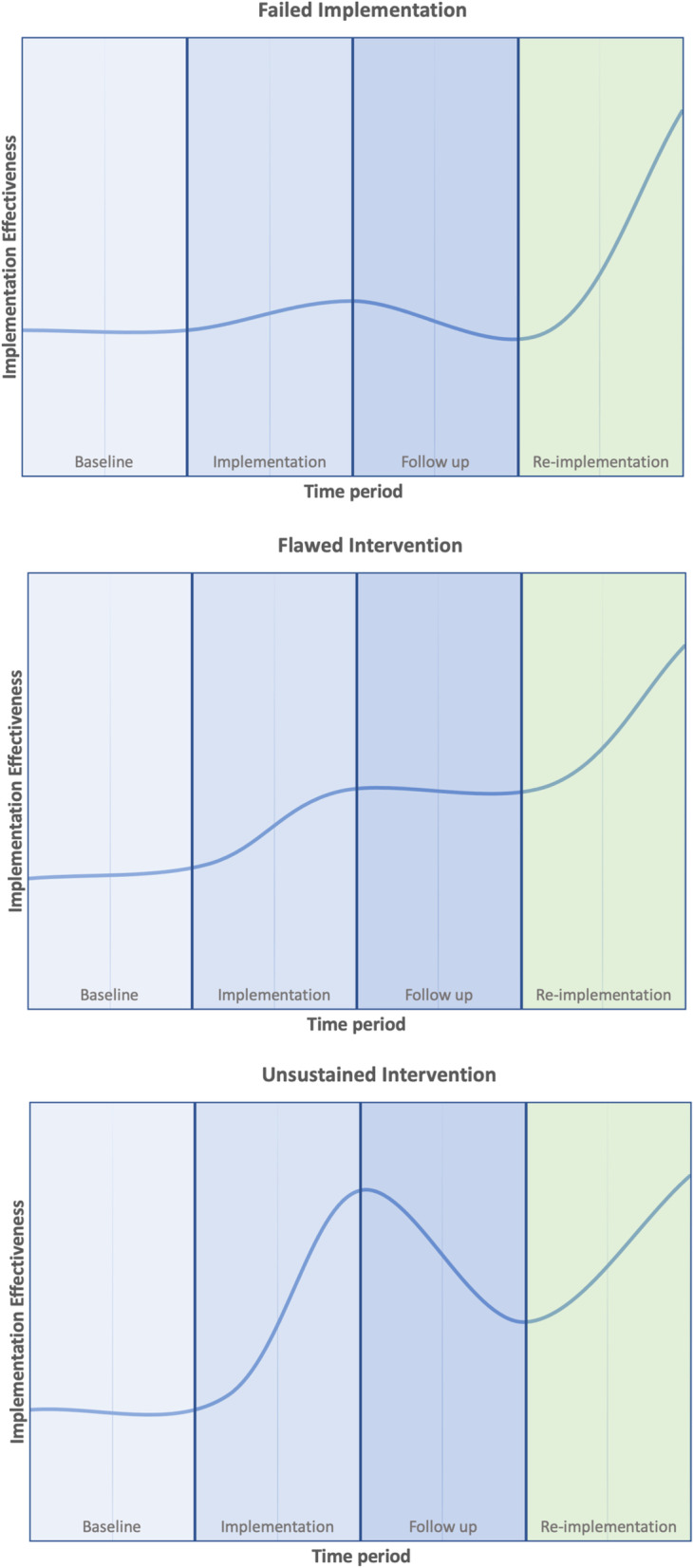
Visual representations of the three types of re-implementation

We identified three distinct circumstances in which re-implementation occurs: (1) failure of the initial implementation process, (2) initial implementation of a flawed intervention, and (3) failure to sustain the intervention (Fig. 2). Multiple types of failure may contribute to the need for re-implementation; for example, a flawed intervention may contribute to a sustainment failure. However, in each case, we were able to identify a dominant failure underlying the need for re-implementation.

#### Type 1: Re-implementation following failed implementation

We identified four examples in the literature in which re-implementation was primarily necessitated by deficiencies in the initial implementation process [25–29]. We termed this subset as re-implementation after failed implementation. Failed implementation is when the initial implementation process fails to result in the intended changes and the adoption of the intervention is low or entirely absent.

The first two cases describe the re-implementation of a surfactant administration protocol and imaging guidelines after discovering low adoption and adherence [25, 26]. In both cases, an evaluation found the main cause of implementation failure was a lack of staff awareness and difficulty accessing the protocol and guidelines. Both cases used the information from the evaluation to inform the re-implementation strategy. They improved awareness through educational meetings and training sessions and moved the guidelines and protocol to a location that increased accessibility. This dual approach was effective in both case examples and improved adoption and adherence [25, 30].

A weak implementation climate and culture can result in a failed implementation, which was the case in the implementation of a barcode medication administration (BCMA) system in the intensive care unit (ICU) [29]. The system had poor adoption by ICU nurses and stopped being used completely eight months after implementation. An evaluation found that nurses were reluctant to use the system due to a policy that tracked medication errors. They rescinded the policy, and their re-implementation process included co-design, training sessions, local champions, and proactive feedback solicitation through BCMA rounds in the ICU. Ultimately, they were unsuccessful in strengthening the implementation climate. They continued to meet significant resistance, with the nurses continuing dual documentation until the ICU converted to an electronic administration system.

The final case example used a multimodal approach through increasing awareness and strengthening the implementation climate with incentives and an enhanced user experience. The Dossier Médical Partagé (DMP), France’s national health information exchange, was initially implemented in 2006 but was stopped 6 months later due to security concerns with the electronic platform [28, 31]. It was re-implemented in 2010 but had very poor adoption, with only 1.5% of the population registered for the DMP 5 years later. One of the primary reasons for low adoption was cultural, with no established culture of sharing medical information among healthcare professionals or with patients [31]. In addition, there was a lack of political support and education for patients. It was re-implemented again in 2016 as part of the French Health Act, with a name change, public awareness campaign, the ability to self-enroll, and a mobile application for patients. In addition, the government offered substantial financial incentives to providers and practices for enrolling their patients in the DMP. These changes to the implementation process were successful, with a significant increase in patients enrolled in the DMP 18 months after re-implementation [27].

#### Type 2: Re-implementation due to a flawed intervention

We identified two examples in the literature where the implementation process was successful, but the intervention failed to achieve the desired outcomes [32, 33]. Both of these cases recognized the need to make major modifications to the intervention before re-implementation. We termed this subset as re-implementation due to a flawed intervention. As previously mentioned, this does not include incremental changes made as part of an improvement cycle. When the intervention undergoes significant modifications, re-implementation is needed to properly engage, train, and launch the intervention to encourage the desired behavior change. The re-implementation process is an ideal time to engage stakeholders and reinvigorate an intervention that is not reaching its full potential.

The intervention for the two cases identified was a chest injury protocol for the emergency department and a comprehensive health assessment in a subacute rehabilitation hospital [32, 33]. The cases had several similarities. They both began with an evaluation and found that the staff was not using the intervention as intended. The re-implementation centered around engaging clinicians to identify challenges and provide input on modifications. In the case of the chest injury protocol, the staff found that it was too complex. The re-implementation included simplifying the protocol and creating new workflows with training sessions to empower nurses and junior staff.

Similarly, in the other case example, the staff found the comprehensive health assessment burdensome and not fully integrated into the clinical team’s practice. Although they could not modify the assessment fields, they did make significant changes to workflows and the electronic platform used to complete the assessment, which was accompanied by a training course for all staff. The changes to the intervention and subsequent re-implementation were effective in both of these examples, with improvements in uptake and meaningful use.

#### Type 3: Re-implementation of an unsustained intervention

We identified five cases in the literature where the initial implementation was successful, but sites did not sustain the changes [34–39]. We termed this subset as re-implementation due to an unsustained intervention. Sustainment is a recognized challenge in implementation, with capacity issues, including workforce, funding, and other resources, as a common reason for failure [3]. It is recognized that the sustainment phase should be dynamic, adapting to the changing environment using rapid learning cycles [1]. However, when the environment has undergone dramatic changes or the intervention was discontinued, these incremental changes may not be sufficient, and transformational changes and re-implementation are necessary.

Slippage, or gradual decline resulting from multiple levels of influence, can occur after the initial success of an intervention [40]. Two case examples identified slippage as the catalyst for re-implementation. The first case example is an evidence-based care bundle for patients undergoing an exploratory laparotomy [34], and the second is implementing a BCMA system in Argentina [35]. In both cases, there was an initial success with a decline over time, with less engagement from the staff and leadership. The re-implementation in both examples relied on engaging closely with clinicians to identify areas for improvement and redesign the intervention. They prioritized sustainment and creating a culture of improvement through training sessions on quality improvement, sharing data, and developing channels for communication with the implementation team. Re-implementation was effective in both cases, and strong leadership engagement was cited as critical to their success.

We identified three cases of programs that abruptly ended due to a resource or funding disruption, which is not an uncommon experience in low and middle-income countries [36]. The first case is a diabetes screening program in Africa that suffered from supply chain disruptions [36]. The second case is a community health worker program in Mozambique, which experienced several challenges in funding related to a lengthy war [37]. The final case is ParticipACTION, a Canadian program promoting physical activity that underwent funding cuts after operating successfully for 30 years [38, 39].

Each case found strong support for re-implementation based on individuals witnessing the intervention’s benefits and previous success. However, some were concerned about future disruptions [36, 37] and how it would fit with other systems that were created to fill gaps when the program ended [37, 39]. In the diabetes screening program, interviews found that the disruption had a cascading effect, hindering team learning and decreasing their self-efficacy to deliver the screening [36]. Their re-implementation strategy included educational programs on handling future supply disruptions and refresher training for teams to increase their self-efficacy when the supplies are available. Although none of the authors commented on the effectiveness of their re-implementation, they all described taking a slow and steady approach while focusing on engaging stakeholders and securing the long-term availability of resources and funding.

## Discussion

Our literature review demonstrates that re-implementation is described and explicitly mentioned in the literature but, to date, has not yet been defined or explored as a key concept in implementation science. We propose that re-implementation offers a more nuanced understanding of the implementation process and occurs when (a) the magnitude of the change is transformational and (b) the intervention had previously been implemented in the same setting. We found it described in various circumstances, with three overarching types identified that relate to the underlying impetus: failed implementation, flawed intervention, and unsustained intervention. Although there are common strategies for re-implementation across all three types, each type also has unique considerations. Defining and understanding the types of re-implementation provide critical information on contextual influences for implementation practitioners to develop their re-implementation strategy and opens up a new area of research in implementation science.

The prevalence of adapting and tailoring to the context in the cases we identified reinforces the influence of context on implementation [41, 42]. However, context may have an even stronger influence in re-implementation because it adds another dimension of factors related to the initial implementation that need to be considered. For example, in addition to standard stakeholder engagement activities, implementation practitioners may need to devote additional time to investigate specific areas. After an implementation failure, seeking input from stakeholders on the challenges encountered during the previous implementation effort is crucial to avoid the causes of failure during re-implementation. If the intervention is flawed, in-depth engagement of stakeholders in the modification process will be essential to create buy-in and incorporate human-centered design principles [43] while considering current practices. Finally, for an unsustained intervention, the focus should be on engaging staff to identify barriers to sustainability and workarounds or other processes created to replace the intervention and accounting for these in the re-implementation strategy.

Through the lens of change management, it becomes apparent how re-implementation may present significant differences from the initial implementation or rapid learning cycles. For example, transformational changes to processes that have already been implemented can be a considerable challenge, with individuals even more resistant when pressured for time, which is common in healthcare settings [14, 44]. This challenge is heightened in the setting of a flawed intervention when re-implementation requires a simultaneous de-implementation of a familiar tool or process. This process of unlearning and relearning creates a tension between prior knowledge and established mental models with the willingness to change [45]. We encountered these challenges during our recent effort re-implementing a flawed intervention and adapted our strategies using re-implementation as a framework, ultimately contributing to our success (Table 2).

**Table 2:** Re-implementing the World Health Organization (WHO) Surgical Safety Checklist

**Intervention**
The WHO Surgical Safety Checklist is a tool to encourage team communication around critical tasks during the perioperative process through a series of conversation prompts and process checks [46, 47]. When used meaningfully, the checklist has reduced surgical morbidity and mortality and improved teamwork [48, 49]. During the initial implementation of the checklist, sites are encouraged to customize it to fit the local context, while not removing any core components or conversation prompts [47]
**Context**
A large tertiary care hospital in Singapore initially implemented an adapted version of the WHO Surgical Safety Checklist over ten years ago. However, most conversation prompts had been removed and it was read by the nurse with no predefined roles for other surgical team members [50]. They had implemented a flawed intervention and gaps in team communication and culture persisted, contributing to several patient safety events
**Re-implementation strategies**
We adapted the strategies from the established implementation guide [47]. Differences included:
• Spending significantly more resources on a comprehensive multimodal evaluation, which included a survey on staff perceptions of the checklist [50]
• Carefully balancing established routines around the checklist with best practices from the implementation guide when modifying the checklist. We conducted a series of observations and feedback sessions to understand the existing practice and surgical culture. We then built upon existing habits, for example, we minimized changes to the timing of when teams use the checklist
• Devoting extra time to modifying and testing the checklist to ensure that stakeholders from all departments were involved and that changes fit the local practice. This was accompanied by frequent meetings with leaders of the surgical departments to collect feedback and address concerns about the updated checklist prior to launch
• Developing training materials focused on the rationale for the changes to the checklist by connecting new items with patient safety events or staff feedback. This approach was designed to overcome resistance expressed by the staff that wished to keep the current checklist and differed from traditional checklist training, which focuses on the rationale for the checklist itself and how to use it [47]

**Fig. 3:**
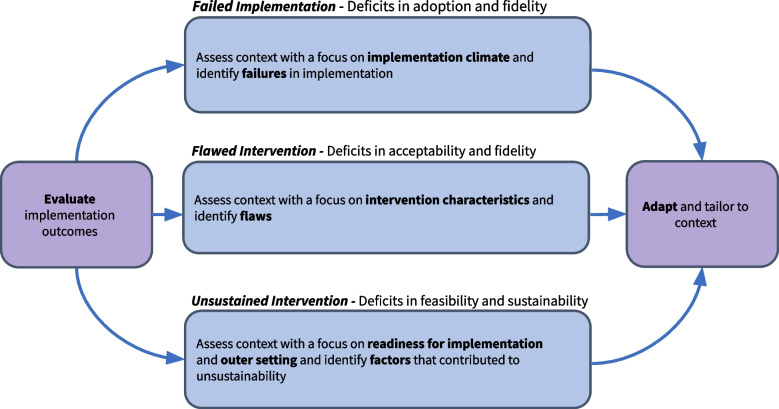
The re-implementation process

In each case reviewed, an evaluation of implementation outcomes triggered re-implementation. There is a benefit to periodically evaluating outcomes during implementation to assess adoption and fidelity and in the sustain phase to detect slippage. There is a decision point following the evaluation: sustainment with incremental changes, re-implement with transformational changes, or de-implement. If the decision is to re-implement, implementation practitioners can first identify the reason for re-implementation, and if it is related to a failure in the implementation process, a flawed intervention, or an unsustained intervention. Recognizing the circumstances for re-implementation provides an area of focus to prevent previous failures from recurring. Reviewing deficits in implementation outcomes can help clarify the circumstances for re-implementation. In the cases we reviewed, there were commonalities in implementation outcomes for each type of re-implementation (Fig. 3). The next step is evaluating the contextual determinants, focusing on areas that are more salient depending on the type of re-implementation. Finally, implementation practitioners can use this information to adapt the intervention and implementation strategy to fit the local environment.

This is the first attempt to systematically describe and define the concept of re-implementation, although it occurs in practice and is explicitly named in the peer-reviewed literature. This article strives to bridge this gap between implementation practice and science. Failing to recognize re-implementation as a concept or classifying transformational changes and intensive re-implementation efforts as continuous implementation impedes further insights, such as differences in resource allocation, training, or messaging during re-implementation planning. We hope our findings spark further conversation about re-implementation and other related topics, such as resilience after implementation failure; the impact of the magnitude of change on implementation; and the integration of management and organization theory into implementation science.

### Limitations

This article has many limitations, the definition and taxonomy may be expanded or refined as re-implementation is further explored as an entity with characteristics and considerations that are unique and distinct from those of initial implementation. We were limited by other areas that have not been clearly defined in implementation science, such as further quantifying the different stages of implementation [3], and the different levels of change in adaptations made during sustainment. Future studies can review and define these concepts, which may overlap and impact our proposed definition of re-implementation.

Our scoping review was carried out with limited resources. It could have been more rigorous, with a full-text review by multiple reviewers and a search that included more databases. Also, we were limited to the depth and breadth of the descriptions of re-implementation found in the literature. There are varying levels of detail in the case examples, so there may have been other strategies or lessons that were part of the re-implementation that the authors did not describe. In addition, there may have been other articles that we did not find in our search. Although we expanded our search to include terms related to re-implementation, others may have described the phenomenon using different terminology. There is also more to learn about others’ re-implementation efforts that have not been published. These limitations emphasize the importance of defining re-implementation, so it can be accurately described and studied. We limited our review to non-policy-related re-implementation because there are significant differences between policy implementation research and implementation science [51]. Our focus was exploring the re-implementation of interventions in health-related contexts, which goes beyond the implementation of a policy or mandate and includes its own unique set of considerations. Future work can consider exploring policy re-implementation and parallels with our findings.

## Conclusion

Defining re-implementation provides a unifying terminology to a phenomenon that occurs in implementation practice but, until now, has not been clearly conceptualized in the literature. By highlighting its existence, we aim to unpack its critical influence on the re-implementation process. Defining and describing re-implementation offer a path forward for unsuccessful or unsustained implementation efforts and a foundation for others as they embark on their re-implementation efforts.

## Supplementary Information

**Additional file 1. **Search strategy. Table that includes databases and search terms.

**Additional file 2. **Re-implementation Cases Identified in the Literature and their Implementation Strategies. Table that includes cases found in the literature with a brief description and corresponding ERIC strategy.

## Data Availability

Data sharing is not applicable to this article as no datasets were generated or analyzed during the current study.

## Abbreviations

BCMA: Barcode medication administration
CFIR: Consolidated Framework for Implementation Research
DMP: Dossier Médical Partagé
ERIC: Expert Recommendations for Implementing Change
ICU: Intensive care unit
PDSA: Plan-Do-Study-Act
RAI-MDS: InterRAI Resident Assessment Instrument–Minimum Data Set
SGH: Singapore General Hospital
